# Burden of gastric cancer attributable to *Helicobacter pylori* in 27 countries from seven geographic regions in 2022

**DOI:** 10.1007/s10120-025-01677-9

**Published:** 2025-10-20

**Authors:** Giulia Collatuzzo, Elton Dajti, Matteo Secco, Franco Bazzoli, Paolo Boffetta, Rocco Maurizio Zagari

**Affiliations:** 1 Department of Medical and Surgical Sciences, University of Bologna, Via Massarenti, 9. 40138 Bologna, Italy; 2 Gastroenterology and Digestive Endoscopy Unit, Michele e Pietro Ferrero Hospital, Verduno, Cuneo Italy; 3 Stony Brook Cancer Center, Stony Brook University, Stony Brook, USA; 4 Department of Family, Population and Preventive Medicine, Renaissance School of Medicine, Stony Brook University, Stony Brook, USA; 5 Gastroesophageal Disease Unit, IRCCS-Azienda Ospedaliero-Universitaria di Bologna, Bologna, Italy

**Keywords:** Gastric cancer, *Helicobacter pylori*, Epidemiology

## Abstract

**Background:**

*Helicobacter (H.) pylori* is the major risk factor of gastric cancer (GC). We aimed to estimate the population attributable fraction (PAF) of GC and the number of new GC cases and deaths for GC attributable to *H. pylori* in different countries worldwide in 2022.

**Methods:**

The PAF was estimated using country-specific pooled prevalence of *H. pylori* in the period 2000–2010 obtained through a systematic review and meta-analysis and a risk ratio of 5.9 for the association between *H. pylori* and GC. The absolute number of new GC cases and deaths for GC attributable to *H. pylori* was calculated using PAF and GC incidence and mortality reported by GLOBOCAN 2022.

**Results:**

The PAF of GC due to *H. pylori* was calculated for 27 countries. The average PAF was 69.7% ranging from 40.7% in Malaysia to 82.3% in South Africa. The PAF was > 70% in all countries in the analysis in Africa, East Mediterranean region and Latin America, apart from Mexico, and in some countries in Western Pacific region (China, Japan and Korea) and Europe (Poland, Spain and Albania). The largest number of GC cases and deaths for GC attributable to *H. pylori* was estimated in China (GC: 252,850, deaths:184,666) and Japan (GC: 92,166, deaths: 31,809).

**Conclusions:**

More than two-thirds of new GC cases in the countries in the analysis were attributable to *H. pylori* in 2022. Our estimates may contribute to better investigate cost-effectiveness of *H. pylori* screening strategies for GC prevention in different countries worldwide.

**Supplementary Information:**

The online version contains supplementary material available at 10.1007/s10120-025-01677-9.

## Introduction

Gastric cancer (GC) is an important cause of morbidity and mortality, and despite the decreasing incidence rate, the total number of cases and deaths due to GC are increasing worldwide [1, 2]. *Helicobacter (H.) pylori* infection is the most important cause of GC leading to gastric adenocarcinoma through chronic gastritis, atrophic gastritis, intestinal metaplasia and dysplasia [3]. The eradication of *H. pylori* is the most effective weapon for the primary prevention of GC [3, 4]. However, cost-effectiveness and antimicrobial resistance are the main concern for *H. pylori* “screen-and-treat” strategies in the general population [3].

The current burden of GC attributable to *H. pylori* in different countries is still unclear. This can be estimated by calculating the population attributable fraction (PAF) of GC for *H. pylori* that defines the proportion of GC cases attributable to *H. pylori* in the general population, i.e., the proportion of cases of GC that could be avoided if *H. pylori* infection was absent or eradicated. The PAF depends on the *H. pylori* prevalence in the general population and the relative risk (RR) of the association between *H. pylori* and GC. Then, multiplying the PAF with the incidence of GC in the population can be calculated the number of new GC cases attributable to *H. pylori* in the general population.

It is well known that prevalence of *H. pylori* infection and incidence of GC vary in different countries and geographic areas [5, 6]. The geographic variation of *H. pylori* prevalence is mainly related to the socioeconomic development with high prevalence in less developed regions and low prevalence in those more developed. In addition, the prevalence of *H. pylori* is dynamic being progressively declining and subjected to the birth–cohort effect [7]. This decline is likely due to several factors including the improvement of socioeconomic status and better sanitation, the increasing use of antibiotics and the widening of indication for eradication therapy [7]. Thus, estimates of proportion and number of new GC cases attributable to *H. pylori* may vary by geographic region and, in particular, by country.

Only few studies have performed a comprehensive analysis to estimate the burden of GC due to *H. pylori* from a global perspective. In addition, previous estimates of the PAF of GC for *H. pylori* have some limitations, in particular related to the calculation of PAF. For example, some studies calculated the PAF retrospectively using the prevalence of *H. pylori* in GC cases rather than prospectively in the general population [8–10], while other studies assumed that the prevalence of *H. pylori* in GC cases can be used as an estimate of PAF [2, 9–13]. Furthermore, other reports used a RR for the association between *H. pylori* and GC derived from retrospective case–control studies, where *H. pylori* was, by necessity, assessed after the development of cancer [14, 15]. *Helicobacter pylori* might be cleared in gastric cancer cases for the presence of atrophic gastritis, so that patients with gastric cancer may be *H. pylori* negative even though they have been infected in the past. Thus, the retrospective design may bias results toward an underestimation of the strength of the association between *H. pylori* and GC.

Improving estimates of the burden of GC due to *H. pylori* in different countries is essential for health policy makers to evaluate cost-effectiveness of *H. pylori* screening and treatment programs for the primary prevention of GC.

The aim of this study was to estimate the PAF of GC for *H. pylori* and the number of new GC cases and deaths for GC attributable to *H. pylori* in different countries worldwide in 2022.

## Methods

The PAF of GC for *H. pylori* was calculated applying the formula originally described by Levin [16], AF = *p* (RR − 1)/(1 + *p* (RR − 1)), where *p* is the prevalence of infection in the general population and RR is the relative risk of GC associated with *H. pylori* infection.

In order to estimate the PAF prospectively, we performed a systematic review and meta-analysis to estimate the country-specific pooled prevalence of *H. pylori* in the adult general population in the period 2000–2010, considering 12–22-year latency between infection and development of GC. This latency period was based on prior evidence that 10 years or more is the best time interval to estimate the association between *H. pylori* infection and GC [17].

The RR for the association between *H. pylori* and GC was assumed to be constant worldwide, and so we used a single RR estimate in the calculation of each PAF. Considering that non-cardia GC represents the majority of the GC cases worldwide (80–90%), we used the RR of 5.9 that was calculated for non-cardia GC through a meta-analysis of prospective case–control studies nested within cohorts where the diagnosis of *H. pylori* infection was made before the development of GC [17]. We applied the same RR to calculate PAF for incidence and mortality and for men and women, assuming no effect of infection on cancer survival and no infection–sex interaction [18].

The absolute number of new GC cases and deaths for GC attributable to *H. pylori* was calculated by multiplying the PAF by the number of new GC cases and deaths for GC estimated from GLOBOCAN 2022 [1]. In particular, GLOBOCAN data were restricted to age 40+, therefore considering population at higher risk of GC [4]. We calculated the PAF of GC for *H. pylori* only for those countries for which was possible to estimate the prevalence of *H. pylori* in the general population in the period 2000–2010. The number of new GC cases and deaths for GC attributable to *H. pylori* was calculated for those countries for which incidence and mortality data on GC from GLOBOCAN 2022 were also available. Countries were grouped according to WHO geographic regions: Africa (2 countries), Americas (7 countries), South-East Asia (1 country), Europe (9 countries), Eastern Mediterranean (2 countries) and Western Pacific (6 countries). As for Americas, we considered data for North America (1 country) and Latin America (6 countries), separately.

### Prevalence of *H. pylori* in the period 2000–2010: systematic review and meta-analysis

The systematic review and meta-analysis were performed according to the PRISMA (Preferred Reporting Items for Systematic Reviews and Meta-Analyses) guideline [19]. We searched MEDLINE via PubMed and Embase up to January 1, 2023, using the following keywords: “*Helicobacter pylori*”, “*H. pylori*”, “*HP*”, “*Campylobacter pylori*” and “*prevalence*”. We did not restrict for language or type of publication. The search strategy is reported in the Table S1. Two authors (ED and MS) did the initial selection based on titles and abstracts. Subsequently, they independently performed a detailed full-text assessment of potentially relevant studies, and any disagreement was solved by a third author (RMZ). Selected studies were included in the review if they reported the prevalence of *H. pylori* in the adult general population in the period 2000–2010, regardless of the year of publication. We excluded studies reporting *H. pylori* prevalence only in children and those that did not meet the inclusion criteria, or if essential information was missing and could not be obtained by the authors. Two authors (ED and MS) extracted independently the following items from each study: country, region, study period, inclusion and exclusion criteria, total number of participants, average age and gender, test for *H. pylori* diagnosis, number and percentage of *H. pylori*-positive subjects. Two authors (ED and MS) independently assessed the quality of the selected studies using the Newcastle–Ottawa Quality Assessment Scale [20] and any disagreement was solved through discussion and, if necessary, arbitration by a third author (RMZ). We calculated the country-specific pooled prevalence of *H. pylori* in the general population with 95% confidence interval (CI) using a random effect model. The heterogeneity among the studies was assessed using the *I*^2^ and the Cochran Q-statistic. All the analyses were performed using STATA 16 [21].

## Results

### Country-specific prevalence of *H. pylori* in the period 2000–2010

The electronic search identified 29,648 records after duplicates were removed, of which 283 full-text articles were assessed for eligibility. Of the 283 articles, 40 met the inclusion criteria and were included in the meta-analysis [22–61]. Figure S1 shows the flowchart of the study selection. The 40 included articles reported the *H. pylori* prevalence in the general population in 27 countries from 7 geographic regions in the period 2000–2010. Two articles provided data from two cohorts, one study from one cohort in Greece and one cohort in Albania [36] and the second one from two cohorts in China [54]; thus, they were included in the meta-analysis two times for a total of 42 studies. Tables S2 and S3 show the characteristics of the included studies and the results of the quality assessment, respectively.

The overall prevalence of *H. pylori* was 52% (108,698 out of 209,035 subjects) ranging from 14.2% in Malaysia to 95% in South Africa; there was high heterogeneity among the studies (*I*^2^ = 99.9%) (Fig. S2). The countries with the highest *H. pylori* prevalence were South Africa (95%), Benin (86.2%), Chile (74.6%), Ecuador (72.2%) and Brazil (70.7%), while USA (27.1%), Norway (26%) and Malaysia (14.2%) showed the lowest *H. pylori* prevalence (Table 1).

**Table 1:** Pooled prevalence of *Helicobacter pylori* in the general population in 27 countries from seven geographic regions in the period 2000–2010

	Studies, *n*	Pooled prevalence of *Helicobacter pylori*, %	95% confidence interval
Africa			
Benin	1	86	81–90
South Africa	1	95	91–97
North America			
USA	1	27	26–28
Latin America			
Brazil	1	71	68–73
Chile	1	75	73–76
Ecuador	1	72	62–80
Guadeloupe (France)	1	54	50–57
Mexico	1	38	33–44
Panama	1	54	43–65
South-East Asia			
Thailand	1	44	37–51
Europe			
Albania	1	53	44–63
Czech Republic	1	42	40–44
Denmark	1	20	19–20
Germany	1	47	46–48
Greece	1	34	25–43
Norway	2	26	24–27
Poland	1	69	65–72
Spain	2	61	58–65
The Netherlands	2	38	37–39
East Mediterranean region			
Iran	5	62	51–72
Tunisia	1	63	57–69
Western Pacific region			
Australia	2	29	27–30
China	5	52	44–59
Japan	2	55	53–56
Korea	3	64	55–72
Malaysia	1	14	13–15
Taiwan	1	39	38–40

### Population attributable fraction of gastric cancer for *H. pylori*

Using the estimates of *H. pylori* prevalence in the general population, we calculated the PAF of GC for *H. pylori* in 27 countries from seven regions in 2022. The average PAF was 69.7% (95% CI 67.8–71.0) ranging from 40.7% in Malaysia to 82.3% in South Africa. The PAF was > 70% in all countries in the analysis in Africa (South Africa: 82.3%, Benin: 80.8%), East Mediterranean region (Tunisia: 75.5%, Iran: 75.2%) and Latin America (Chile: 78.6%, Ecuador: 77.9%, Brazil: 77.7%, Guadalupe: 72.6%, Panama: 72.6%) apart from Mexico (65.1%), and in some countries in the Western Pacific region (Korea: 75.8%, Japan: 72.9%, China: 71.8%) and Europe (Poland:77.2%, Spain:74.9% and Albania:72.2%). The lowest PAF was found in Australia (58.7%), USA (57%), Norway (56%), Denmark (49.5%) and Malaysia. Table 2 shows the estimates of PAF of GC for *H. pylori* by countries.

**Table 2:** Population attributable fraction of gastric cancer for *Helicobacter pylori* infection in 27 countries from seven geographic regions in 2022

	Population attributable fraction, %	95% confidence interval, %
Africa		
Benin	80	79.9–81.5
South Africa	82.3	81.7–82.6
North America		
USA	57.0	56.0–57.8
Latin America		
Brazil	77.7	76.9–78.2
Chile	78.6	78.2–78.8
Ecuador	77.9	75.2–79.7
Guadeloupe (France)	72.6	71.0–73.6
Mexico	65.1	61.8–68.3
Panama	72.6	67.8–76.1
South-East Asian Region		
Thailand	68.3	64.5–71.8
Europe		
Albania	72.2	68.3–75.5
Czech Republic	67.3	66.2–68.3
Denmark	49.5	48.2–49.5
Germany	69.7	69.3–70.2
Greece	62.5	55.1–67.8
Norway	56.0	54.0–57.0
Poland	77.2	76.1–77.9
Spain	74.9	74.0–76.1
The Netherlands	65.1	64.4–76.1
Eastern Mediterranean Region		
Iran	75.2	71.4–77.9
Tunisia	75.5	73.6–77.2
Western Pacific Region		
Australia	58.7	57.0–59.5
China	71.8	68.3–74.3
Japan	72.9	72.2–73.3
Korea	75.8	72.9–77.9
Malaysia	40.7	38.9–42.4
Taiwan	65.6	65.0–66.2

### Number of new gastric cancer cases and deaths for gastric cancer attributable to *H. pylori*

Using our estimates of AF and the estimates of GC incidence and mortality reported by GLOBOCAN 2022 [1], we calculated the absolute number of new GC cases (Table 3) and deaths for GC (Table 4) attributable to *H. pylori* in 26 countries in 2022. Estimates for Taiwan were not provided because data from GLOBOCAN 2022 were lacking.

**Table 3:** Number of new cases of gastric cancer attributable to *Helicobacter pylori* infection in 26 countries from seven geographic regions in 2022

	Number of new cases of gastric cancer^a^			Number of new cases of gastric cancer attributable to *Helicobacter pylori*		
	Total	Males	Females	Total	Males	Females
Africa						
Benin	378	254	124	305	205	100
South Africa	1784	1080	704	1468	889	579
North America						
USA	24,668	14,976	9692	14,060	8536	5524
Latin America						
Brazil	22,300	14,402	7898	17,327	11,190	6135
Chile	4902	3327	1575	3853	2615	1238
Ecuador	2586	1513	1073	2015	1179	836
Guadeloupe (France)	124	70	54	90	51	39
Mexico	9001	4784	4217	5860	3114	2745
Panama	472	291	181	342	211	131
South-East Asia						
Thailand	3907	2147	1760	2669	1467	1202
Europe						
Albania	701	460	241	506	332	174
Czech Republic	1228	730	498	826	491	335
Denmark	725	473	252	359	234	125
Germany	13,917	8263	5654	9703	5761	3942
Greece	1857	1168	689	1161	730	431
Norway	512	339	173	287	190	97
Poland	6874	4431	2443	5305	3420	1885
Spain	7084	4224	2860	5308	3165	2143
The Netherlands	1814	1088	726	1180	708	472
East Mediterranean Region						
Iran	16,895	10,671	6224	12,711	8028	4683
Tunisia	508	312	196	384	236	148
Western Pacific Region						
Australia	2776	1718	1058	1629	1008	621
China	352,084	243,507	108,577	252,850	174,875	77,975
Japan	126,366	83,816	42,550	92,166	61,132	31,034
Korea	28,823	19,511	9312	21,855	14,794	7061
Malaysia	1431	969	462	582	394	188

^a^Data derived by GLOBOCAN 2022. The values represent absolute numbers. Estimated from Taiwan are not included because incidence data on gastric cancer from GLOBOCAN 2022 were not available

**Table 4:** Number of deaths for gastric cancer attributable to *Helicobacter pylori* infection in 26 countries from seven geographic regions in 2022

	Number of deaths for gastric cancer^a^			Number of deaths for gastric cancer attributable to *Helicobacter pylori*		
	Total	Males	Females	Total	Males	Females
Africa						
Benin	324	217	107	261	175	86
South Africa	1548	859	689	1274	707	567
North America						
USA	10 650	6451	4199	9747	6070	3677
Latin America						
Brazil	17 569	11 335	6234	13 646	8804	4842
Chile	3876	2620	1256	3047	2060	987
Ecuador	2104	1223	881	1639	953	686
Guadeloupe (France)	96	59	37	70	43	27
Mexico	6819	3619	3200	4437	2355	2082
Panama	355	219	136	258	159	99
South-East Asia						
Thailand	2970	1638	1332	2029	1119	910
Europe						
Albania	555	366	189	401	264	137
Czech Republic	923	547	376	621	368	253
Denmark	465	306	159	231	152	79
Germany	8643	5178	3465	6026	3610	2416
Greece	1243	788	455	776	492	284
Norway	320	187	133	180	105	75
Poland	5311	3411	1900	4098	2632	1466
Spain	5008	3047	1961	3752	2283	1469
The Netherlands	1172	722	450	763	470	293
East Mediterranean Region						
Iran	13 515	8848	4667	10 168	6657	3511
Tunisia	439	269	170	331	203	128
Western Pacific Region						
Australia	1309	841	468	769	494	275
China	257 141	179 964	77 177	184 666	129 241	55 425
Japan	43 611	27 529	16 082	31 809	20 079	11 730
Korea	8386	4914	3472	6359	3726	2633
Malaysia	1158	821	337	471	334	137

^a^Data derived by GLOBOCAN 2022. The values represent absolute numbers. Estimated from Taiwan are not included because data on mortality for gastric cancer from GLOBOCAN 2022 were not available

A total of 454,802 new cases of GC and 287,829 deaths for GC could be attributable to *H. pylori* infection across 26 countries in 2022.

The highest numbers of new GC cases and deaths for GC due to *H. pylori* were seen in China (GC: 252,850, deaths: 184,666) and Japan (GC: 92,166, deaths: 31,809), followed by Korea for the number of new GC cases (GC: 21,855) and Brazil (deaths: 13,646) and Iran (deaths: 10,168) for the number of deaths for GC *H. pylori*-related. In Latin America, Brazil showed the highest number of new GC cases due to *H. pylori* (GC: 17,327). In Europe, the number of GC cases and GC deaths attributable to *H. pylori* was remarkable in Germany (GC: 9703, deaths: 6026), Spain (GC: 5308, deaths: 3752) and Poland (GC: 5305, deaths: 4098), while GC cases and deaths for GC *H. pylori*-related were less than 1000 in Albania, Czech Republic, Denmark, Greece, Norway and The Netherlands. In the East Mediterranean region, the number of new GC cases due to *H. pylori* was much higher in Iran (GC: 12,711) than in Tunisia (GC: 384). Finally, in Africa there was the lowest number of new GC cases (n. 1773) and GC deaths (n. 1535) attributable to *H. pylori,* despite the high *H. pylori* prevalence and AF of GC for *H. pylori*. The number of new GC cases (Table 3) and deaths for GC (Table 4) attributable to *H. pylori* was about two times higher in men than women in all countries.

## Discussion

This study provides estimates of the burden of GC attributable to *H. pylori* infection in 27 countries from seven geographic regions in 2022. Population attributable fractions are based on country-specific pooled prevalence of *H. pylori* in the general population in the period 2000–2010 estimated through a systematic review and meta-analysis. Using estimates of PAF and the estimates of GC incidence and mortality provided by GLOBOCAN [1], we calculated the number of new GC cases and deaths for GC attributable to *H. pylori* infection in the different countries.

Our data confirm the major role played by *H. pylori* in the burden of GC [6, 8], in particular in China, Japan, Korea, Brazil and Iran. We found that the average PAF of GC for *H. pylori* was 69.7% in 2022. This estimate is only slightly lower than those previously reported in 2008 (77.3%) [8] and in 2012 (79.3%) [9]. Obviously, the PAF varied across different countries in line with the variation of *H. pylori* prevalence, which is in agreement with other pooled analyses [62, 63].

Notably, we found that in China, Japan and Korea the PAF of GC for *H. pylori* was higher than 70%. Previous estimates of the PAF in these countries were not consistent [11, 13, 14, 62–64]. In the study by Ji, the PAF of GC for *H. pylori* was 58%, 30.9% and 35.2% in Japan, China and Korea, respectively [14]. Similarly, Han et al. reported low estimates of PAF in East Asia including China (45.6%), Japan (57.1%) and Korea (40.5%), even though the sub-group analysis of studies with a follow-up time > 10 years showed a PAF of non-cardia GC of 71.2% [63]. Similarly, a large case–control study including about 500,000 adults reported in China a PAF of 78.5% for non-cardia gastric cancer and 62.1% for cardia gastric cancer [64]. In line with this study, we provide further evidence that more than two-third of new GC cases in Japan, China and Korea are due to *H. pylori* infection. In particular, *H. pylori* infection may have caused about 255,000 cases of GC and 182,000 deaths for GC in China and 92,000 cases of GC and 32,000 deaths for GC in Japan in 2022.

The number of GC cases and deaths for GC due to *H. pylori* was different in different countries, also due to differences in the total number of incident GC cases that is the result of different incidence rates of gastric cancer and population size. The total number of incident GC was very high in China and Japan [2], and this would partially explain the largest number of GC cases and deaths due to *H. pylori* in these countries. This also results in the different number of GC cases due to *H. pylori* that occurred in different countries within Latin America, East Mediterranean Region and Europe. For example, in the East Mediterranean Region, although the PAF was the same, the number of gastric cancer attributable to *H. pylori* was much higher in Iran than in Tunisia due to the higher incidence rate of gastric cancer and larger population size, which in turn results in a greater total number of incident GC cases, in Iran.

In Africa, *H. pylori* prevalence and PAF of GC for *H. pylori* were > 80%, but the number of GC cases and deaths attributable to *H. pylori* was very low due to the low incidence of GC in Africa. This supports the historical concept of the “African Enigma”, based on the low GC rates despite the persistently high prevalence of *H. pylori* [65].

Current guidelines suggest focusing on GC prevention through population-based *H. pylori* screen-and-treat strategies [3]. Notably, *H. pylori* screening and treatment programs present many challenges, including high costs, complex study protocols and design and possible increase in antimicrobial resistance in the general population [3]. However, in Asia *H. pylori* screening and treatment programs seem to be cost-effective [3, 66]. Kowada et al. reported that *H. pylori* screening performs better in terms of cost-effectiveness than endoscopy program in Japan, and that it is most effective in asymptomatic adults aged 50 or older [66]. Notably, in order to further reduce the GC burden, Japan and Korea added *H. pylori* screening and eradication to the health insurance policy in the early 2010s [67]. Indeed, our finding further supports the suggestion that *H. pylori* screening and treatment programs in Asia, in particular in China and Japan, would be cost-effective for reducing the high burden of GC in the general population. Whether an *H. pylori* screen-and-treat strategy is cost-effective in non-Asian countries is still under debate [3, 68]. The 2022 Council of the European Union Recommendation on Cancer Screening recommended the implementation of screen-and-treat strategies in those countries with high GC incidence [69]. We estimated the number of GC cases and deaths for GC preventable through *H. pylori* eradication in several European countries, and we showed that the burden of GC *H. pylori-*related preventable by a screening strategy would be substantially high in Germany, Spain and Poland than in other European countries. Indeed, our results may help health policy makers to better explore cost-efficacy of population-based *H. pylori* screening programs in some countries in Europe. Furthermore, the development of solid prediction models and risk stratification would allow to integrate *H. pylori* eradication programs to the already implemented cancer screening [3, 70–72]. In addition to eradication treatment, strategies for the prevention of *H. pylori* infection including better sanitation and hygiene status, cleaner water supply and, eventually, vaccination could help reducing the burden of GC, in particular in resource-limited settings.

Our study has several strengths. We estimated the burden of GC attributable to *H. pylori* using country-specific data and accurate statistical methods. In particular, we calculated the PAF prospectively using the country-specific prevalence of *H. pylori* infection in the general population estimated through a systematic review and meta-analysis. Furthermore, we considered a 10–20-year latency between *H. pylori* infection and the development of GC. Such latency was not always considered [8–10, 13, 73, 74]; given the secular decline of *H. pylori* prevalence in most countries [7], this would have resulted in an underestimation of the PAF of GC for *H. pylori*. Finally, we adopted the estimates of GC incidence and mortality provided by GLOBOCAN 2022 to calculate the number of new GC cases and deaths for GC attributable to *H. pylori* infection [1].

This study has also some limitations. The first limitation is the paucity of data on *H. pylori* prevalence for certain geographical areas, such as Africa, East Mediterranean region and South-East Asia, that may affect the accuracy of the estimates of PAF and also prevented us from providing estimates of attributable cases and deaths by region and globally. We did not provide a global estimate of the prevalence of *H. pylori* infection and of the burden of GC attributable to it, as other authors have done [6, 8–10], because of the many assumptions needed to derive global estimates. Rather, we decided to focus on the countries with data of acceptable quality, thus giving priority to validity over generalizability. The relatively large number of countries in the analysis, including several major countries, on the other hand, provides clinical and public health relevance of our findings. Some studies included also children/adolescents in the analysis of *H. pylori* prevalence, and we could not restrict the estimates of *H. pylori* prevalence to the adult population. Studies including both adults and children/adolescents reported lower estimates of *H. pylori* prevalence, and this may likely contribute to an underestimation of PAF in some countries (USA, Mexico, Czech Republic, the Netherland, etc.). In addition, we could not overcome limitations related to the accuracy of the diagnostic methods for *H. pylori* infection that may vary across studies. Furthermore, in order to obtain data on the prevalence of H*. pylori* infection in the general population for the highest number of countries worldwide, we included studies conducted across a wide range of years (from 2000 to 2010). We cannot exclude that variations in the prevalence of *H. pylori* and PAF may reflect not only geographic differences but also the timing of surveys and the age of population (birth–cohort effect).

Moreover, we assumed that RR between *H. pylori* and GC was constant worldwide, assuming no differences in the strength of this association by geographical area. While this is a common statistical approach, it may not represent the reality as pathogen- and individual-related factors may play a role in the association between infection and GC; for example, interactions with smoking or dietary factors could modify the carcinogenetic effect of *H. pylori* [63]; *Helicobacter pylori* strains also influence carcinogenicity, with CagA positive ones being known for their stronger association with cancer [26, 32, 56]. However, other studies adopted values of RR that were estimated by pooling retrospective case–control studies, likely leading to an underestimation of the strength of the association between *H. pylori* and GC due to the possibility that GC cases lose infection, and therefore due to an underestimation of the PAF [11, 13–15]. We did not provide data by GC anatomical sub-site, i.e., for cardia and non-cardia GC. It is well that known that *H. pylori* have different strengths of association with cardia and non-cardia GC [64]. However, on a global scale most of GC are non-cardia that are strongly related to *H. pylori* infection. Finally, we applied the same RR to calculate PAF for both incidence and mortality assuming no effect of infection on cancer survival. However, some retrospective studies including a relatively small group of patients have suggested that *H. pylori* infection may have a positive effect on overall survival of GC patients [75]. Thus, assuming equal risk between incidence and mortality, we might have slightly overestimated the number of deaths for GC attributable to *H. pylori.*

In conclusion, this study provides the most recent comprehensive analysis of the burden of GC attributable to *H. pylori* from a global perspective. We found that *H. pylori* remains the most important cause of GC leading to more than two-thirds of new GC cases and deaths for GC worldwide.

The burden of GC due to *H. pylori* varies among different countries, even though they belong to the same geography region and have similar *H. pylori* prevalence. Therefore, country-specific data on the PAF and number of new GC cases and deaths for GC attributable to *H. pylori* are essential in the perspective of *H. pylori* screening programs for the prevention of GC. Our findings represent an important contribution to further investigating the cost-effectiveness of such strategies in different countries worldwide. Given the changing trends in *H. pylori* prevalence and GC incidence, studies providing accurate and updated estimates of country-specific burden of GC attributable to *H. pylori* are needed in future.

## Supplementary Information

Below is the link to the electronic supplementary material.

Supplementary file1 (PDF 1276 KB)

## Data Availability

The data are available from the corresponding author on reasonable request and completion of necessary privacy and ethical approvals.
