# Supplementary material for: Burden of gastric cancer attributable to Helicobacter pylori in 27 countries from seven geographic regions in 2022
Source: Gastric Cancer. 2025 Oct 20;29(1):16–26. doi: 10.1007/s10120-025-01677-9 (PMC12830462; doi:10.1007/s10120-025-01677-9)

Supplementary Materials

BURDEN OF GASTRIC CANCER ATTRIBUTABLE TO *HELICOBACTER PYLORI* IN 27 COUNTRIES FROM SEVEN GEOGRAPHIC REGIONS IN 2022

Giulia Collatuzzo[1], Elton Dajti[1], Matteo Secco[2], Franco Bazzoli[1], Paolo Boffetta[1,3,4], Rocco Maurizio Zagari[1,5].

[1] Department of Medical and Surgical Sciences, University of Bologna, Bologna, Italy. [2] Gastroenterology and Digestive Endoscopy Unit, Michele e Pietro Ferrero Hospital, Verduno, Cuneo, Italy. [3] Stony Brook Cancer Center, Stony Brook University, Stony Brook, USA.[4] Department of Family, Population and Preventive Medicine, Renaissance School of Medicine, Stony Brook University, Stony Brook, USA. [5] Gastroesophageal Disease Unit, IRCCS - Azienda Ospedaliero-Universitaria di Bologna, Bologna, Italy.

Corresponding Author

Rocco Maurizio Zagari, MD, Gastroesophageal Disease Unit, IRCCS – Azienda Ospedaliero-Universitaria di Bologna. Department of Medical and Surgical Sciences, University of Bologna. Via Massarenti, 9. 40138 Bologna, Italy. Email: roccomaurizio.zagari@unibo.it

Table S1. Electronich search strategy (up to 1.01.2023).

Electronic database	Search string	Results, n.
PUBMED	((“HP” OR “Helicobacter Pylori” OR “H. Pylori” OR “Campylobacter pylori” OR "Helicobacter pylori" [MeSh Terms]) AND “prevalence”)	25094
EMBASE	((‘HP’ OR ‘Helicobacter pylori’ OR ‘H pylori’ OR ‘Campylobacter pylori’) AND ‘prevalence’)	10275

Fig. S1. Flow chart of systematic literature search.

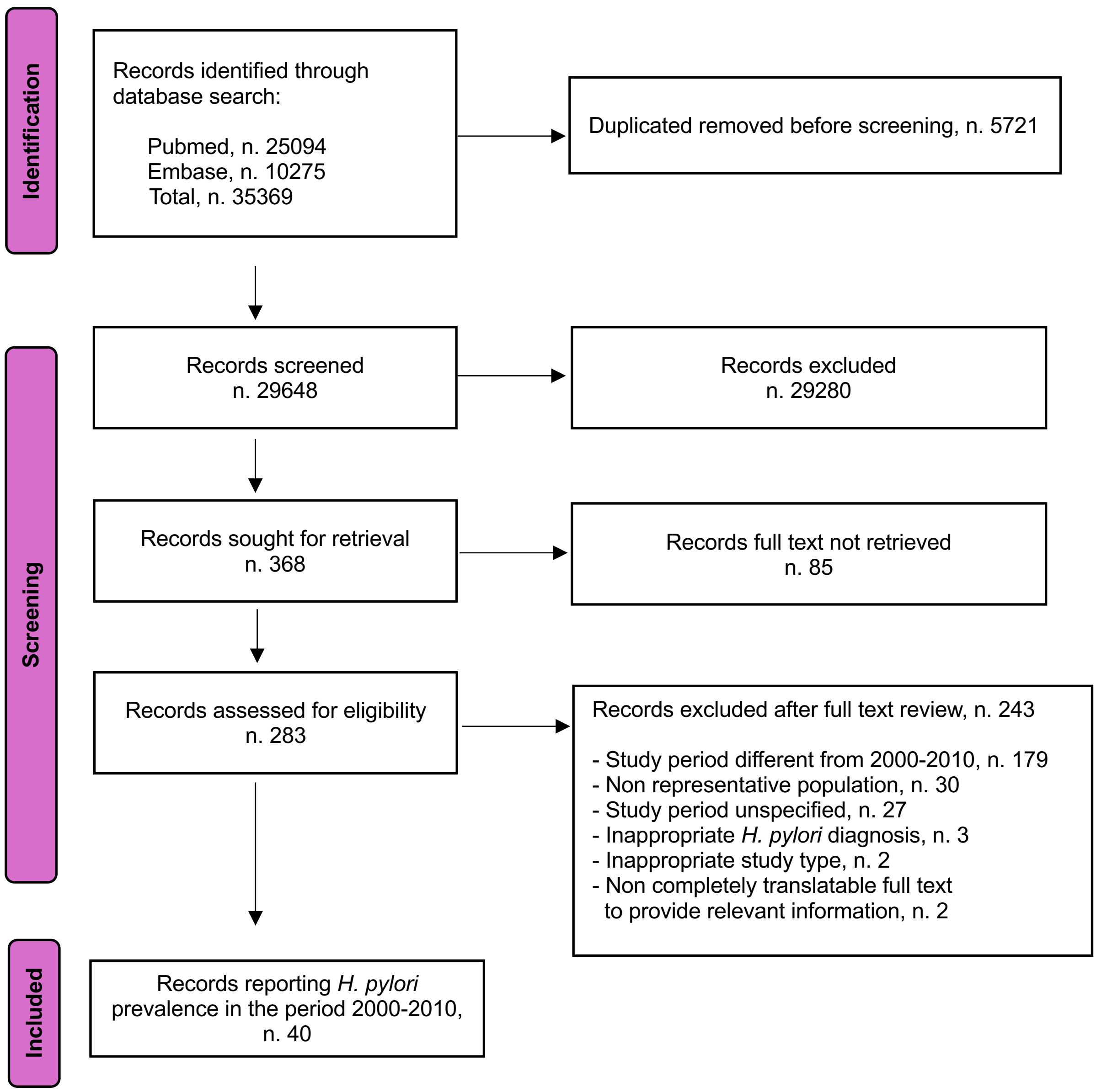

Table S2. Characteristics of the studies included in the meta-analysis for *Helicobacter pylori* prevalence in the period 2000-2010.

Reference	Country	Region	Study period	Subjects, n.	Age range (years)	*H. pylori* positive subjects, n.	*H.pylori* prevalence %	95% Confidence Interval	*H. pylori* diagnosis
Aguemon, B. D.[22]	Benin	Africa	2003-2004	247	16-74	213	86.2	70.0-80.9	Serology
Dube, C.[23]	South Africa	Africa	2008	228	25 ->60	216	94.7	83.3-90.3	SAT
Santos, I. S.[24]	Brazil	Latin America	2006-2007	1001	18-45	708	70.7	67.9-73.6	UBT
Ferreccio, C.[25]	Chile	Latin America	2003	2615	>17	1951	74.6	72.9-76.2	Serology
Sasaki, T.[26]	Ecuador	Latin America	2007	90	21- 82	65	72.2	63.0-81.5	SAT
Sasaki T.[27]	Panama	Latin America	2007	74	21-82	40	54	42.7-65.4	SAT
Weill, F. X.[28]	Guadeloupe (France)	Latin America	2000	854	18-70	459	53.7	51,7-58,8	Serology
Cardenas, V. M.[29]	Mexico	Latin America	2004	288	>1	110	38.2	31.7-44.6	SAT
Alavi, S. M.[30]	Iran	Eastern Mediterranean	2004-2005	96	30-60	55	57.3	47.4-67.2	Serology
Farshad, S.[31]	Iran	Eastern Mediterranean	2005-2007	226	18-83	94	41.6	35.2-48.0	RUT
Jafarzadeh, A.[32]	Iran	Eastern Mediterranean	2004	138	17-60	101	73.2	65.8-80.6	Serology
Jafarzadeh, A.[33]	Iran	Eastern Mediterranean	2005	200	20-60	135	67.5	61.0-74.0	Serology
Nouraie, M.[34]	Iran	Eastern Mediterranean	2005	2326	18-65	1605	69	67.1-70.9	Serology

Mansour, K. B.[35]	Tunisia	Eastern Mediterranean	2006-2007	250	25-55	158	63.2	57.2-69.2	Serology
Katsanos, K. H. [36]	Albania	Europe	2005-2008	101	20 – 50	54	53.5	43.7-63.2	Histology
Katsanos, K. H.[36]	Greece	Europe	2005-2008	101	20-50	34	33.7	24.5-42.9	Histology
Bures, J et al. [37]	Czech Republic	Europe	2001	2509	5-100	1046	41.7	39.8-43.6	UBT
Kornerup L.S.[38]	Denmark	Europe	2002 - 2012	53633	>18	10552	19.7	19.3-20.0	UBT
Holleczek B.[39]	Germany	Europe	2000-2002	9940	>18	4708	47.4	46.4-48.3	Serology
Loffeld L. J. R. F.[40]	Netherland	Europe	1993-2002	8190	4-99	3201	39.1	38.0-40.1	Histology
Van Blankestein M.[41]	Netherland	Europe	2005	1551	17-80	491	31.7	29.4-34.0	Serology
Asfeldt, A. M.[42]	Norway	Europe	2004	916	18-85	348	38	34.9-41.1	Histology
Breckan, R. K.[43]	Norway	Europe	2004-2005	1736	18-85	368	21.2	19.3-23.1	SAT
Celinski, K.[44]	Poland	Europe	2000-2003	585	19-89	402	68.7	65.0-72.5	Serology
Baena Diez, J. M.[45]	Spain	Europe	1999-2001	208	>20	133	63.9	46.4-58.4	Serology
Sanchez Ceballos F.[46]	Spain	Europe	2004-2006	481	4-82	290	60.3	55.9-64.7	UBT
Cardenas, V. M.[47]	USA	North America	1999–2000	7462	3-70	2022	27.1	26.1-28.1	Serology
Ang, T. L.[48]	Thailand	South-East Asian	2007–2008	179	40-80	78	43.6	36.3-50.8	SAT
Moujaber, T.[49]	Australia	Western Pacific	2002	1811	>15	325	17.9	13.7-16.6	Serology
Windsor, H. M.[50]	Australia	Western Pacific	2003-2004	520	2-90	395	76	72.3-79.6	UBT
Chen, J.[51]	China	Western Pacific	2003	1006	20-92	565	56.2	44.5-49.6	Serology

Cheng, H.[52]	China	Western Pacific	2003	1232	2-79	577	46.8	44.0-49.5	UBT
Shi, R.[53]	China	Western Pacific	2004 – 2005	1371	5-100	851	62.1	59.5-64.6	Serology, UBT
Zhang, D. H.[54]	China	Western Pacific	2006	503	40-79	208	41.4	37.1-45.7	SAT
Zhang, D. H.[54]	China	Western Pacific	2006	526	40 -79	268	51	46.7-55.2	Histology
Fujimoto, Y.[55]	Japan	Western Pacific	2002	3819	17-84	2116	55.4	46.2-58.9	Serology
Hirai, I.[56]	Japan	Western Pacific	2007	186	40 -63	75	40.3	33.3-47.4	SAT
Kim, N.[57]	Korea	Western Pacific	2006	20154	16 ->70	12173	60.4	59.7-61.1	Serology, histology
Sung.K.C.[58]	Korea	Western Pacific	2001 – 2002	58981	30 - >60	41818	70.9	70.5-71.3	Serology, SAT, histology
Yim, J.Y.[59]	Korea	Western Pacific	2005	8020	≥20	4780	59.6	58.5-60.74	Serology
Sasidharan, S.[60]	Malaysia	Western Pacific	2000-2002	5370	10-70	763	14.2	13.2-15.1	Serology
Lin, Y. L.[61]	Taiwan	Western Pacific	2004 – 2006	9311	> 40	3650	39.2	38.3-40.2	RUT

H. pylori: Helicobacter pylori; CI: confidence interval; SAT: stool antigen test; UBT: urea breath test; RUT: rapid urease test.

Table S3. Quality assessment of selected studies.

Author (reference)	Representativeness of the cases*
Aguemon B.D. [22]	B
Dube C. [23]	B
Santos I.S. [24]	B
Ferreccio C. [25]	A
Sasaki T. [26]	B
Sasaki T. [27]	B
Weill F. [28]	B
Cardenas V.M. [29]	B
Alavi S.M. [30]	B
Farshad S. [31]	B
Jafarzadeh A. [32]	B
Jafarzadeh A. [33]	B
Nouraie M. [34]	B
Mansour K.B. [35]	B
Katsanos K.H. [36]	B
Bures J. [37]	A
Kornerup L.S. [38]	B
Holleczek B. [39]	B
Loffeld R.J.L.F. [40]	B
van Blankenstein M. [41]	B
Asfeldt A.M. [42]	A
Breckan R.K. [43]	A
Celinsky K. [44]	B
Baena Diez J.M. [45]	B
Sanchez Ceballos F. [46]	B
Cardenas V.M. [47]	A
Ang T.L. [48]	A
Moujaber T. [49]	B
Windsor H.M. [50]	B
Chen J. [51]	B
Cheng H. [52]	B
Shi R. [53]	B
Zhang D.H. [54]	B
Fujimoto Y. [55]	B
Hirai I. [56]	B
Kim N. [57]	B
Sung K.C. [58]	B
Yim J.Y. [59]	B
Sasidharan S. [60]	B
Lin Y.I. [61]	B

*Representativeness of the cases:
A: Truly representative of the average prevalence of *Helicobacter pylori* infection in the community.
B: Somewhat representative of the average prevalence of *Helicobacter pylori* infection in the community.
C: Selected group of users; e.g. nurses, volunteers.
D: No description of the derivation of the cohort.

Fig. S2. Forest plot of the prevalence of *Helicobacter pylori* in the general population in 2000-2010 by country.

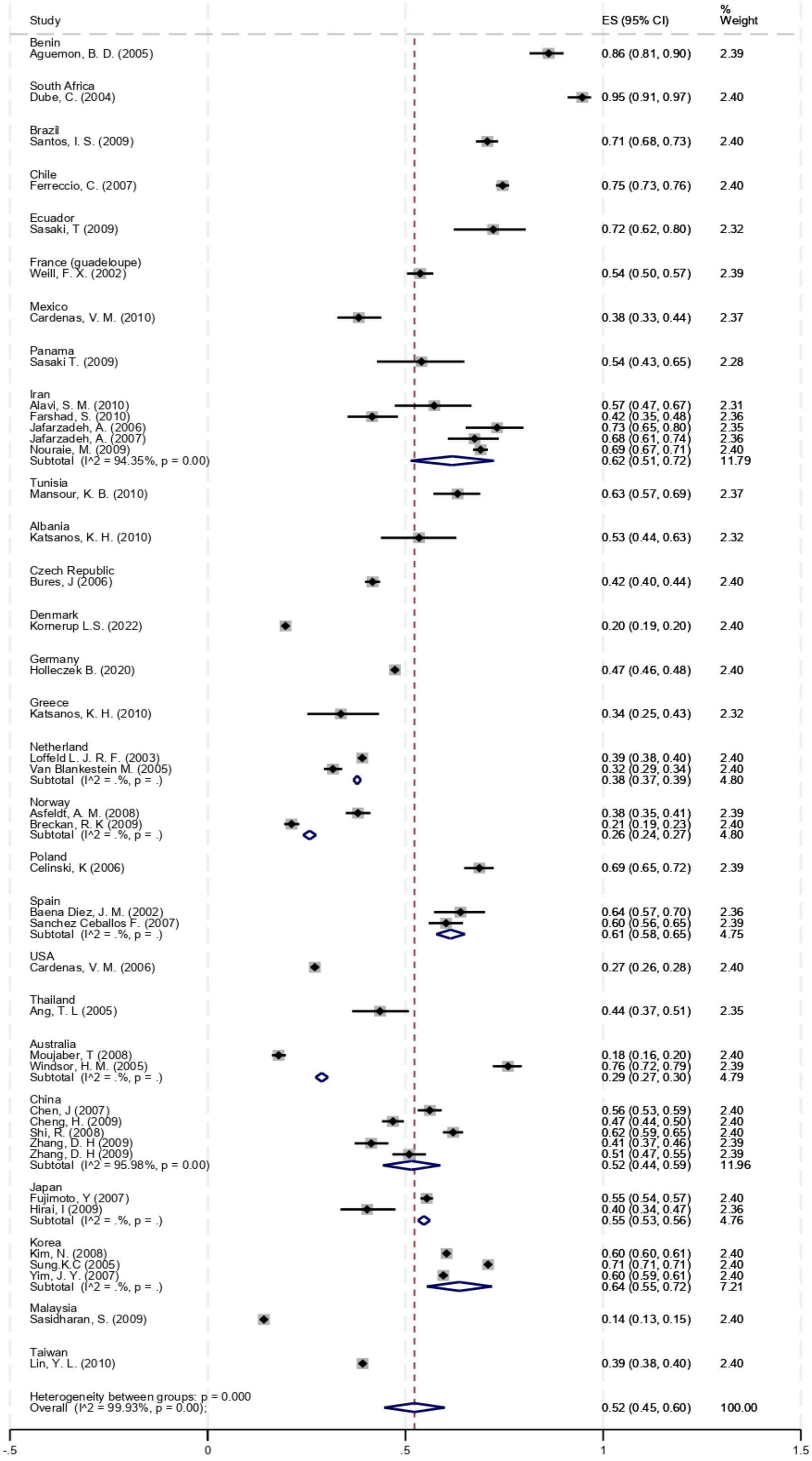

Supplement: Supplementary file 1 — Supplementary file1 (PDF 1276 KB) [file 10120_2025_1677_MOESM1_ESM.pdf]
